# Digital microvascular reactivity does not decline with impaired renal function in chronic kidney disease

**DOI:** 10.1186/s12882-019-1484-x

**Published:** 2019-07-30

**Authors:** Lulu Wang, Xiaoqin Huang, Weichun He, Wenjin Liu, Junwei Yang

**Affiliations:** 1 Center for Kidney Disease, Second Affiliated Hospital of Nanjing Medical University, 262# North Zhongshan Road, Nanjing, 210003 China; 2 Department of Radiology, University of Washington, 850 Republican Street, Seattle, 98109 USA

**Keywords:** Reactive hyperemia index, Endothelial dysfunction, Renal function, CKD, Cardiovascular diseases

## Abstract

**Background:**

The reactive hyperemia index (RHI), measured by peripheral arterial tonometry (PAT), is a novel measurement of endothelial function and has been proven to be valuable in cardiovascular risk stratification in several populations. The current study aims to explore its relation to renal function and its association with traditional cardiovascular risk factors in patients with chronic kidney disease (CKD).

**Methods:**

Subjects with non-dialysis dependent CKD were recruited and 252 of them had a successful PAT test. In addition to general demographic and medical information, carotid-femoral pulse wave velocity (cfPWV), carotid-radial pulse wave velocity (crPWV) and augmentation index (AIx) were recorded.

**Results:**

The mean age of the study population was 57.7 (±14.7) years and 155 (61.5%) were males. The average RHI was 1.92 (±14.7) with no difference noted between males and females. There was no statistically significant correlation between RHI and eGFR (r = − 0.107, *p* = 0.089) or urine protein-to-creatinine ratio (r = 0.036, *p* = 0.570). With adjustment for age and sex, RHI was associated with systolic blood pressure (BP) (β = 0.006, *p* = 0.001), diastolic BP (β = 0.008, *p* = 0.010), heart rate (β = − 0.007, *p* = 0.015) crPWV (β = 0.037, *p* = 0.022) and AIx (β = 0.006, *p* = 0.001), but not with cfPWV or any other conventional risk factors analyzed. Systolic BP remained the only predictor for RHI in the stepwise regression analysis.

**Conclusions:**

RHI did not decline with reduced renal function in CKD patients and had a modest association with traditional cardiovascular risk factors. Further studies are warranted to determine if RHI could predict cardiovascular outcome in CKD patients.

## Introduction

Endothelial dysfunction is a key early step in the development and progression of atherosclerosis [1]. It precedes the occurrence of various clinically overt cardiovascular diseases (CVD). The assessment of endothelial dysfunction has been proven to be valuable for risk prediction in the general population, as well as in subjects with a high risk profile [2, 3]. Multiple methods for the detection of endothelial dysfunction have been established since the 1980s [4]. Generally, noninvasive assessment is preferred over invasive procedures. However, even flow-mediated dilation (FMD), the most widely used noninvasive method, has been criticized for its high dependency on experienced technicians and lack of reproducibility [5, 6]. In recent decades, the application of peripheral arterial tonometry (PAT) in the measurement of endothelial function has been widely adopted in clinical research. This technique is barely operator-dependent and the result is analyzed automatically. Multiple lines of evidence have validated its value in cardiovascular risk stratification among high-risk subjects [7, 8]. However, there remains a lack of insight into the underlying pathophysiologic meaning of its cardiovascular risk prediction ability, especially given its noted difference from FMD [9].

Chronic kidney disease (CKD) is a potent and independent risk factor for cardiovascular morbidity and mortality. Patients with CKD are more likely to die of cardiovascular events than to progress into end-stage renal disease [10]. This increased cardiovascular risk starts at the early stage of CKD and deteriorates as renal function declines. The cause of this higher risk in CKD patients is multifaceted and involves the combined effects of various traditional and nontraditional risk factors. Endothelial dysfunction has been considered an important nontraditional factor that contributes to the increased cardiovascular risk in the context of CKD. Therefore, it is expected that the PAT test could serve as an important tool for cardiovascular risk stratification in these patients. However, relevant studies are scarce. Important questions yet to be answered include: 1. Does the microvascular function evaluated by PAT decline with impaired renal dysfunction? and 2. What are the relations of digital microvascular function to traditional cardiovascular risk factors? In light of extensive evidence linking reduced renal function to impaired vascular health, we hypothesize that microvascular reactivity measured by PAT deteriorates as renal function declines and is associated with other established cardiovascular risk factors in CKD patients. We tested this hypothesis in the current study in a group of non-dialysis-dependent CKD patients.

## Methods

### Study design and participants

This cross-sectional study was approved by the Institutional Ethics Committee of the Second Affiliated Hospital of Nanjing Medical University. All subjects provided written informed consent before enrollment. Patients aged ≥18 years with a clinical diagnosis of CKD (non-dialysis dependent) who were referring to our center were invited to participate in the study. The diagnosis of CKD conformed to the KDIGO (Kidney Disease: Improving Global Outcomes) guidelines and was confirmed by the research staff before enrollment [11]. We excluded those 1) with a previous history of renal transplantation, 2) with acute infection or any other unstable conditions when the physicians considered the patient unsuitable for immediate participation.

### Peripheral arterial tonometry

Digital microvascular function was evaluated by a PAT device (EndoPAT 2000; Itamar Medical, Caesarea, Israel) as reported previously [12]. The tests were performed in the morning (usually between 8:00 am and 9:00 am) in a quiet test room with the patients in the fasting state. Antihypertensive medications and short-acting nitrates were not allowed within 2 h, and long-acting nitrates were restrained for 12 h before measurement. The test room temperature was set at 22–25 °C. Two finger probes were placed on the index finger of each hand. After 6 min of baseline measurement, pulsatile arterial flow was occluded through inflation of a blood pressure cuff on the test arm for 5 min. The cuff was inflated starting at a pressure of 250 mmHg and increasing until a complete occlusion was achieved as judged by the PAT signal or to a maximum of 300 mmHg. The signal was recorded for another 5 min after occlusion. The reactive hyperemia index (RHI) was calculated according to the algorithm in the system automatically, which is the ratio of amplitude of the signal after cuff deflation divided by those before cuff inflation, indexed to the contralateral arm. Higher RHI indicates better microvascular reactivity.

### Pulse wave velocity (PWV)

Carotid-femoral PWV (cfPWV) and carotid-radial PWV (crPWV) were determined in the same morning as PAT using the Complior Analyzer device (Artech Medical, France). PWV is considered the gold standard for evaluating arterial elasticity, and cfPWV and crPWV are widely used measurements of central and peripheral arterial stiffness, respectively [13]. With the patients in the supine position, three probes were applied to a place that had a palpable pulse of the carotid, femoral and radial artery, respectively. The transit time was averaged over ten consecutive recordings using the intersecting tangent algorithm as recommended. Carotid–femoral and carotid–radial distances were measured and multiplied by 0.8 for calculation of PWV. With the carotid probe, the device also recorded the central pressure waveform directly. The augmentation index (AIx) was calculated as the ratio of the augmented pressure (amplitude difference of the first and second wave) to the central pulse pressure.

### General data collection

Patients were interviewed by research staff to obtain their general demographic and medical information. When necessary, medical records were also used for data acquisition or validation. Height and weight were measured to the nearest 0.5 cm and 0.1 kg, respectively. Blood pressure (BP) was measured using an automated oscillometric device (Omron HEM-7130; Omron Healthcare Co. Ltd., Kyoto, Japan) after the subject had rested in a seated position for at least 10 min. Three measurements were taken consecutively at 1-min intervals. The last two measurements were averaged for final analysis. Previous history of cardiovascular disease referred to any history of acute myocardial infarction, ischemic or hemorrhagic stroke, coronary heart disease other than myocardial infarction, chronic heart failure, atrial fibrillation and other types confirmed by the research staff. Estimated glomerular filtration rate (eGFR) was calculated according to the CKD-EPI formula [14]. CKD stage 1–2 was defined as eGFR ≥60 ml/min/1.73m^2^, stage 3 was defined as eGFR < ml/min/1.73m^2^ and ≥ 30 ml/min/1.73m^2^, and stage 4–5 was defined as eGFR < 30 ml/min/1.73m^2^.

### Statistical analysis

Numerical variables are presented as mean ± sd or median (IQR) based on their distribution. Dichotomous variables are presented as frequency (percentage). RHI was not logarithmized for the analysis as it was in some previous publications because it was normally distributed in our study. Comparisons among different CKD stages were conducted using one-way ANOVA (analysis of variance), and Bonferroni’s method was used for post hoc between-group comparisons. The association between RHI and renal function / proteinuria was analyzed using Pearson’s correlation analysis. For the analysis of the relationships, our sample had 80% power to detect a weak correlation with r as low as 0.175, and 90% power to detect a correlation with r = 0.202. The association of RHI with each individual cardiovascular risk factor was evaluated using a generalized linear model with adjustment for age and sex. To determine the independent predictors for RHI, those significantly associated with RHI in univariate analysis were included in a multivariate stepwise regression model. All analyses were performed using SPSS 19.0 (IBM SPSS, Chicago, IL). A two-tailed *p* value < 0.05 was considered statistically significant.

## Results

From December 2016 to December 2018, a total of 262 patients were enrolled in the study. PAT was performed in all patients and was successful in 252 subjects. There were 10 cases of failed PAT tests due to poor PAT signals. The current analysis includes only those with a validated PAT results (*N* = 252).

The general characteristics of the study subjects are presented in Table 1. There were 155 (61.5%) males and 97 (38.5%) females. The average age was 57.7 (±14.7) years and the mean body mass index (BMI) was 25.9 (±4.1) kg/m^2^. The leading causes of renal failure were glomerulonephritis (33.7%) and diabetic nephropathy (31.3%). Cardiovascular diseases were present in 23.8% of the patients. The mean eGFR of the study population was 51.7 (±33.5) ml/min/1.73m^2^. The average RHI was 1.92 (±14.7), and those with CKD stage 1–2 had a lower RHI (i.e., worse vascular reactivity) compared to those with CKD stage 3 (1.82 ± 0.53 vs 2.03 ± 0.58; *p* = 0.03). No difference was noted for stage 1–2 vs 4–5 or stage 3 vs 4–5 (stage 1–2 vs 4–5: 1.82 ± 0.53 vs 1.92 ± 0.53, *p* = 0.700; stage 3 vs 4–5: 2.03 ± 0.58 vs 1.92 ± 0.53, *p* = 0.533).

**Table 1:** General Characteristics of the Study Subjects

	All (*N* = 252)	CKD Stage 1–2 *N* = 90	CKD Stage 3 *N* = 80	CKD Stage 4–5 *N* = 82
Age, yrs	57.7 ± 14.7	50.7 ± 13.8	61.8 ± 13.4	61.5 ± 14.0
Male	155 (61.5%)	61 (67.8%)	46 (57.5%)	48 (58.5%)
BMI, kg/m^2^	25.9 ± 4.1	26.9 ± 4.3	25.4 ± 3.9	25.2 ± 4.0
Current smoker	72 (28.6%)	35 (38.9%)	23 (28.8%)	14 (17.1%)
Etiology				
*Diabetic nephropathy*	79 (31.3%)	23 (25.6%)	26 (32.5%)	30 (36.6%)
*Hypertensive nephropathy*	23 (9.1%)	6 (6.7%)	9 (11.3%)	8 (9.8%)
*Glomerulonephritis*	85 (33.7%)	45 (50.0%)	28 (35.0%)	12 (14.6%)
*Others*	25 (9.9%)	7 (7.8%)	6 (7.5%)	12 (14.6%)
*Unknown*	40 (15.9%)	9 (10.0%)	11 (13.8%)	20 (24.4%)
CVD	60 (23.8%)	12 (13.3%)	25 (31.3%)	23 (28.0%)
Diabetes	116 (46.0%)	37 (41.1%)	36 (45.0%)	43 (52.4%)
Statins	105 (41.7%)	38 (42.2%)	36 (45.0%)	31 (37.8%)
eGFR, ml/min/1.73m^2^	51.7 ± 33.5	88.9 ± 22.1	45.5 ± 8.9	16.8 ± 7.4
Urine PCR, mg/mmol	172.1 (47.9–413.1)	134.4 (37.4–273.0)	122.7 (33.1–391.1)	284.9 (127.8–606.3)
Hemoglobin, g/L	118.1 ± 24.7	132.9 ± 19.1	121.7 ± 21.6	98.4 ± 19.5
Albumin, g/L	36.5 ± 7.3	36.1 ± 8.5	36.4 ± 6.9	37.1 ± 6.2
Total cholesterol, mmol/L	4.91 ± 1.73	5.13 ± 1.66	4.85 ± 1.94	4.71 ± 1.57
Triglycerides, mmol/L	2.12 ± 1.67	2.53 ± 2.15	1.77 ± 1.26	2.02 ± 1.29
HDL-C, mmol/L	1.16 ± 0.43	1.15 ± 0.41	1.24 ± 0.44	1.09 ± 0.44
LDL-C, mmol/L	3.05 ± 1.35	3.24 ± 1.49	2.98 ± 1.36	2.91 ± 1.13
Systolic BP, mm Hg	136.5 ± 19.9	129.7 ± 17.5	137.6 ± 18.0	143.0 ± 21.8
Diastolic BP, mm Hg	84.2 ± 11.0	85.3 ± 9.9	83.4 ± 10.9	83.7 ± 12.3
Heart Rate, bpm	74.0 ± 12.3	76.5 ± 12.6	72.1 ± 11.7	72.9 ± 12.0
RHI	1.92 ± 0.55	1.82 ± 0.53	2.03 ± 0.58	1.92 ± 0.53

Abbreviations: *BMI* body mass index, *BP* blood pressure, *CVD* cardiovascular disease, *eGFR* estimated glomerular filtration rate, *PCR* protein-to-creatinine ratio, *HDL-C* high density lipoprotein cholesterol, *LDL-C* low density lipoprotein cholesterol, *RHI* reactive hyperemia index

We first analyzed the correlation of RHI to renal function and urine protein-to-creatinine ratio (uPCR) using Pearson’s correlation analysis (Table 2). Unexpectedly, there was no statistically significant correlation between RHI and eGFR (r = − 0.107, *p* = 0.089) or RHI and uPCR (r = 0.036, *p* = 0.570). RHI seemed to be inversely correlated to eGFR (worse renal function with better RHI) with a marginal *p* value. Considering that patients with glomerulonephritis may undergo very different clinical management involving immunomodulators compared to those with other etiologies, we performed a separate analysis including only those with hypertensive or diabetic nephropathy. There was still no association between RHI and eGFR or uPCR in this subgroup.

**Table 2:** Univariate Correlation of RHI to Renal Function and Proteinuria

	r	*p*
eGFR	−0.107	0.089
Urine PCR^a^	0.036	0.570

^a^log-transformed

Abbreviations: *eGFR* estimated glomerular filtration rate, *PCR* protein-to-creatinine ratio, *RHI* reactive hyperemia index

We further explored the association of RHI and cardiovascular risk factors in CKD patients (Table 3). With adjustment for age and sex, RHI did not show significant associations with most of the risk factors analyzed. It was positively associated with systolic BP (β = 0.006, *p* = 0.001) and diastolic BP (β = 0.008, *p* = 0.010), and was negatively associated with heart rate (β = − 0.007, *p* = 0.015). Analyzing artery stiffness markers, higher RHI was associated with increased crPWV (β = 0.037, *p* = 0.022) but not with cfPWV (β = 0.011, *p* = 0.308). It also showed a positive association with AIx (β = 0.006, p = 0.001). When all the above factors associated with RHI were included in a stepwise regression analysis, only systolic BP (β = 0.006, *p* < 0.001) remained an independent predictor for RHI in the final model.

**Table 3:** Association between RHI and Cardiovascular Risk Factors Adjusted for Age and Sex

	β	*p*
Age^a^	0.002	0.330
BMI	0.007	0.448
Current Smoker	0.068	0.446
Diabetes	−0.023	0.749
CVD	−0.061	0.495
Statins	−0.085	0.235
Total Cholesterol	0.007	0.723
Triglycerides	−0.020	0.365
HDL-C	0.017	0.840
LDL-C	0.017	0.520
Systolic BP	**0.006**	**0.001**
Diastolic BP	**0.008**	**0.010**
Heart Rate	**−0.007**	**0.015**
cfPWV	0.011	0.308
crPWV	**0.037**	**0.022**
AIx	**0.005**	**0.010**

Bold entries indicate significance

Abbreviations: *AIx* augmentation index, *BMI* body mass index, *BP* blood pressure, *cfPWV* carotid-femoral pulse wave velocity, *crPWV* carotid-radial pulse wave velocity, *CVD* cardiovascular disease, *HDL-C* high density lipoprotein cholesterol, *LDL-C* low density lipoprotein cholesterol, *RHI* reactive hyperemia index

^a^Adjusted only for sex

## Discussions

In the current study, we explored the relationship between microvascular reactivity, as measured by PAT, and renal function, as well as the association of microvascular reactivity with general cardiovascular risk factors in a group of CKD patients. Our major findings include the following: First, RHI, the measurement of digital microvascular reactivity, did not decline with impaired renal function; second, RHI was not related to most traditional cardiovascular risk factors in the patients, though a positive significant association was found with systolic BP (i.e., higher systolic BP was associated with better microvascular reactivity).

The lack of a significant association of lower RHI with impaired renal function is an unexpected but interesting result. CKD has been a well-known independent risk factor for cardiovascular diseases [15]. Abnormalities of vascular function, including endothelial dysfunction, have been reported to be prevalent in CKD patients and are associated with adverse cardiovascular outcomes [16–19]. A variety of CKD-associated factors contribute to impaired endothelial function in these patients, including elevated BP, insulin resistance, systemic inflammation, oxidative stress, high serum phosphate, HDL dysfunction, uremic toxin and hemodialysis procedure [20–26]. In light of the enrichment of risk factors associated with declined renal function, a graded association between endothelial dysfunction and reduced GFR is expected and has been validated in several previous studies. Yilmaz et al. studied 406 patients with nondiabetic CKD and found a significant positive correlation between FMD and eGFR [18]. Similar results have also been noted by others [27–29]. Although these studies used FMD to evaluate endothelial function, very few studies have explored the relationship between PAT-derived RHI and renal function in the CKD population. Hirata et al. measured RHI in a group of patients who underwent coronary angiography, including 383 subjects with non-dialysis-dependent CKD. In contrast to our result, they found that RHI significantly decreased with increased CKD severity (defined as a combination of eGFR and proteinuria) [30]. However, it should be noted that the CKD population they studied was patients with suspected coronary artery disease and with at least one coronary risk factor, which essentially represents a subpopulation of patients with cardiovascular disease. Meanwhile, their study population was much older than ours, with an average age of 72.0 (±9.0) years. In such a diseased population, more severe coronary artery injury, which results from more impaired endothelial function, might result in increased CKD severity. Hence, the graded association they found between RHI and CKD severity could possibly be a reflection of the link between RHI and coronary endothelial dysfunction [8]. Interestingly, in a subsequent report from the same group, renal function did not differ between subjects with low RHI and those with high RHI among 150 patients who underwent PCI with reduced eGFR [31].

The lack of an association between RHI and eGFR could be related to the complex underlying mechanisms of the digital hyperemia response, which is not yet fully understood. Given the existence of arteriovenous anastomoses, nitric oxide plays a limited role in the regulation of vascular tone in the digital circulation [32]. PAT-derived RHI is, therefore, not fully dependent on endothelial function. Prior investigations have demonstrated that inhibition of nitric oxide synthesis only abolishes less than 50% of the digital hyperemia response in vivo, whereas it completely eliminates FMD [33, 34]. The endothelial-independent mechanism underlying RHI may not be affected by impaired renal function. In line with our data, Moerland et al. compared RHI between 6 CKD patients and 12 healthy subjects and found no statistical difference between the two groups, with RHI in the patients even tending to be higher than in the healthy controls (2.9 ± 1.4 vs 1.8 ± 0.5, *p* = 0.15), [35]. In fact, we also noted that patients with CKD stage 3 had a better RHI compared to those with CKD stage 1–2, and there was also a nonsignificant trend toward increased RHI with declined renal function in our study, which is possibly due to the increased BP in more advanced CKD patients, as our subsequent analysis revealed that RHI was independently and positively associated with systolic BP. Besides the unclear contribution of PAT-related biological pathways, other factors may also have confounded the effect. For example, fluid retention and increased atherosclerosis of digital vessel walls could have impacted the measurement of RHI and might be potential explanations for the lack of relation between RHI and eGFR.

We also analyzed the correlation of RHI to cardiovascular risk factors in the current study and found that RHI was not correlated to most of them in the CKD patients. In the age- and sex-adjusted analysis, lower RHI was associated with higher systolic and diastolic BP, higher heart rate, higher crPWV and higher Aix. Higher systolic BP remained the sole independent predictor for lower RHI in the stepwise regression analysis. The direction of this association is counterintuitive since hypertension has been a traditional atherosclerosis risk factor. A possible explanation for this finding could be that elevated BP is associated with larger baseline pulse amplitude, which is the denominator for the calculation of RHI and hence resulted in lower RHI. Notably, the association between systolic BP and RHI was modest, with a small β of 0.006 (every 10 mmHg increase in BP was associated with an increase in RHI of only 0.06). This indicates that the variance of RHI in these patients can be explained by the traditional risk factors to a very limited extent and it should be determined mainly by nonconventional factors that were not studied in our study.

The lack of association of RHI with general cardiovascular risk factors in the CKD population seems to differ from the findings in the general population. To date, PAT-derived RHI has been studied in two large population-based cohorts. In the Third Generation Cohort of the Framingham Heart Study (*N* = 1957, average age of 40 years), RHI was found to be lower in males and in those with higher BMI, higher total/HDL cholesterol, diabetes and smoking habitus [36]. Unexpectedly, reduced RHI was associated with younger age and the use of lipid-lowering drugs. No independent association was noted between RHI and either BP. In the Brazilian Longitudinal Study of Adult Health (ELSA-Brasil) cohort study, an independent and positive association between RHI and systolic BP was found in 1535 community-based participants (mean age of 52 years) [37], similar to our findings. Unlike in the Framingham study, a negative association was found between age and RHI. Other factors associated with lower RHI in the cohort included male sex, higher BMI, higher total/HDL cholesterol, use of antihypertensive medication and prevalence of cardiovascular diseases. The study also measured cfPWV and found that it was not related to RHI. It should be noted that the stepwise regression models for predicting RHI in the two studies both had a small R^2^(0.159 and 0.14), which validates our speculation that RHI is more affected by factors yet to be identified than these traditional cardiovascular risk factors. Unfortunately, neither of the two studies reported renal function or its association with RHI.

Given the lack of association of RHI with general cardiovascular risk factors in CKD patients, an important question beyond the current study would be whether RHI is capable of predicting cardiovascular outcome in these patients as it does in other disease populations [7, 38, 39]. In the aforementioned study by Hirata et al., RHI predicted cardiovascular event risk in those with CKD [30]. However, these patients were chosen from a group of patients with suspected coronary artery disease, among whom the prognosis predicting value of RHI has been validated. Exploring the association of RHI with cardiovascular outcome in a more general CKD population is warranted. It should be acknowledged that the lack of association of RHI with conventional cardiovascular risk factors does not necessarily mean that RHI is not associated with cardiovascular outcome since impaired RHI could represent a novel pathophysiologic pathway to cardiovascular events.

Our study has several limitations that should be pointed out for appropriate interpretation of the results. First, the cross-sectional observational design cannot establish any temporal relationship and precludes causal inrference. RHI and eGFR were both measured at a single time point in the study so we were not able to capture the temporal changes. Given the progressive nature of CKD, further study with a prospective design would be preferred to test if RHI declines with renal function over time. Second, the current study only included those with established CKD and we were, therefore, unable to compare RHI between subjects with and without CKD. As discussed previously, such a comparison has been conducted in a small study and the results revealed no difference [35]. Third, our study was restricted to the Chinese CKD population, and the results might not be generalized to other ethnic groups. Lastly, patients willing to participate in such a study are usually healthier than those who are not. This is a potential bias toward better microvascular reactivity in the current study.

## Conclusions

In conclusion, digital microvascular function, as measured by PAT-derived RHI, did not decline with reduced renal function in a group of non-dialysis dependent CKD patients. RHI was not independently associated with most of the conventional cardiovascular risk factors in the patients while it was positively and modestly associated with systolic BP. Further studies are warranted to determine if RHI could predict cardiovascular outcome in CKD patients.

## Data Availability

All data generated or analysed during this study are included in this published article and its supplementary information files.

## Abbreviations

Aix: Augmentation index
BMI: Body mass index
BP: Blood pressure
cfPWV: carotid-femoral pulse wave velocity
CKD: Chronic kidney disease
crPWV: carotid-radial pulse wave velocity
CVD: Cardiovascular diseases
eGFR: estimated glomerular filtration rate
FMD: Flow-mediated dilation
PAT: Peripheral arterial tonometry
PWV: Pulse wave velocity
RHI: Reactive hyperemia index
uPCR: urine protein-to-creatinine ratio

## References

[1] Gimbrone MA, Garcia-Cardena G (2016). Endothelial cell dysfunction and the pathobiology of atherosclerosis. Circ Res.

[2] Shechter M, Shechter A, Koren-Morag N, Feinberg MS, Hiersch L (2014). Usefulness of brachial artery flow-mediated dilation to predict long-term cardiovascular events in subjects without heart disease. Am J Cardiol.

[3] Ras RT, Streppel MT, Draijer R, Zock PL (2013). Flow-mediated dilation and cardiovascular risk prediction: a systematic review with meta-analysis. Int J Cardiol.

[4] Ludmer PL, Selwyn AP, Shook TL, Wayne RR, Mudge GH, Alexander RW, Ganz P (1986). Paradoxical vasoconstriction induced by acetylcholine in atherosclerotic coronary arteries. N Engl J Med.

[5] Deanfield J, Donald A, Ferri C, Giannattasio C, Halcox J, Halligan S, Lerman A, Mancia G, Oliver JJ, Pessina AC (2005). Endothelial function and dysfunction. Part I: methodological issues for assessment in the different vascular beds: a statement by the working group on endothelin and endothelial factors of the European Society of Hypertension. J Hypertens.

[6] Charakida M, Masi S, Luscher TF, Kastelein JJ, Deanfield JE (2010). Assessment of atherosclerosis: the role of flow-mediated dilatation. Eur Heart J.

[7] Rubinshtein R, Kuvin JT, Soffler M, Lennon RJ, Lavi S, Nelson RE, Pumper GM, Lerman LO, Lerman A (2010). Assessment of endothelial function by non-invasive peripheral arterial tonometry predicts late cardiovascular adverse events. Eur Heart J.

[8] Bonetti PO, Pumper GM, Higano ST, Holmes DR, Kuvin JT, Lerman A (2004). Noninvasive identification of patients with early coronary atherosclerosis by assessment of digital reactive hyperemia. J Am Coll Cardiol.

[9] Allan RB, Delaney CL, Miller MD, Spark JI (2013). A comparison of flow-mediated dilatation and peripheral artery tonometry for measurement of endothelial function in healthy individuals and patients with peripheral arterial disease. European journal of vascular and endovascular surgery : the official journal of the European Society for Vascular Surgery.

[10] Keith DS, Nichols GA, Gullion CM, Brown JB, Smith DH (2004). Longitudinal follow-up and outcomes among a population with chronic kidney disease in a large managed care organization. Arch Intern Med.

[11] Eknoyan G, Lameire N, Eckardt K, Kasiske B, Wheeler D, Levin A, Stevens P, Bilous R, Lamb E, Coresh J (2013). KDIGO 2012 clinical practice guideline for the evaluation and management of chronic kidney disease. Kidney Int.

[12] Wang L, Liu W, Yu Y, Jiang L, Yang J (2018). Increased circulating bioactive C-type natriuretic peptide is associated with reduced heart rate variability in patients with chronic kidney disease. BMC Nephrol.

[13] Laurent S, Cockcroft J, Van Bortel L, Boutouyrie P, Giannattasio C, Hayoz D, Pannier B, Vlachopoulos C, Wilkinson I, Struijker-Boudier H (2006). Expert consensus document on arterial stiffness: methodological issues and clinical applications. Eur Heart J.

[14] Levey AS, Stevens LA, Schmid CH, Zhang YL, Castro AF, Feldman HI, Kusek JW, Eggers P, Van Lente F, Greene T (2009). A new equation to estimate glomerular filtration rate. Ann Intern Med.

[15] Sarnak MJ, Levey AS, Schoolwerth AC, Coresh J, Culleton B, Hamm LL, McCullough PA, Kasiske BL, Kelepouris E, Klag MJ (2003). Kidney disease as a risk factor for development of cardiovascular disease: a statement from the American Heart Association councils on kidney in cardiovascular disease, high blood pressure research, clinical cardiology, and epidemiology and prevention. Circulation.

[16] Townsend RR, Wimmer NJ, Chirinos JA, Parsa A, Weir M, Perumal K, Lash JP, Chen J, Steigerwalt SP, Flack J (2010). Aortic PWV in chronic kidney disease: a CRIC ancillary study. Am J Hypertens.

[17] Zoungas S, Cameron JD, Kerr PG, Wolfe R, Muske C, McNeil JJ, McGrath BP (2007). Association of carotid intima-medial thickness and indices of arterial stiffness with cardiovascular disease outcomes in CKD. American journal of kidney diseases : the official journal of the National Kidney Foundation.

[18] Yilmaz MI, Saglam M, Carrero JJ, Qureshi AR, Caglar K, Eyileten T, Sonmez A, Cakir E, Yenicesu M, Lindholm B (2008). Serum visfatin concentration and endothelial dysfunction in chronic kidney disease. Nephrology, dialysis, transplantation : official publication of the European Dialysis and Transplant Association - European Renal Association.

[19] Stam F, van Guldener C, Becker A, Dekker JM, Heine RJ, Bouter LM, Stehouwer CD (2006). Endothelial dysfunction contributes to renal function-associated cardiovascular mortality in a population with mild renal insufficiency: the Hoorn study. Journal of the American Society of Nephrology : JASN.

[20] Dogra G, Irish A, Chan D, Watts G (2006). Insulin resistance, inflammation, and blood pressure determine vascular dysfunction in CKD. American journal of kidney diseases : the official journal of the National Kidney Foundation.

[21] Nowak KL, Chonchol M, Ikizler TA, Farmer-Bailey H, Salas N, Chaudhry R, Wang W, Smits G, Tengesdal I, Dinarello CA (2017). IL-1 inhibition and vascular function in CKD. Journal of the American Society of Nephrology : JASN.

[22] Yilmaz MI, Saglam M, Caglar K, Cakir E, Sonmez A, Ozgurtas T, Aydin A, Eyileten T, Ozcan O, Acikel C (2006). The determinants of endothelial dysfunction in CKD: oxidative stress and asymmetric dimethylarginine. American journal of kidney diseases : the official journal of the National Kidney Foundation.

[23] Stevens KK, Denby L, Patel RK, Mark PB, Kettlewell S, Smith GL, Clancy MJ, Delles C, Jardine AG (2017). Deleterious effects of phosphate on vascular and endothelial function via disruption to the nitric oxide pathway. Nephrology, dialysis, transplantation : official publication of the European Dialysis and Transplant Association - European Renal Association.

[24] Shroff R, Speer T, Colin S, Charakida M, Zewinger S, Staels B, Chinetti-Gbaguidi G, Hettrich I, Rohrer L, O'Neill F (2014). HDL in children with CKD promotes endothelial dysfunction and an abnormal vascular phenotype. Journal of the American Society of Nephrology : JASN.

[25] Kim HY, Yoo TH, Hwang Y, Lee GH, Kim B, Jang J, Yu HT, Kim MC, Cho JY, Lee CJ (2017). Indoxyl sulfate (IS)-mediated immune dysfunction provokes endothelial damage in patients with end-stage renal disease (ESRD**)**. Sci Rep.

[26] Meyer C, Heiss C, Drexhage C, Kehmeier ES, Balzer J, Muhlfeld A, Merx MW, Lauer T, Kuhl H, Floege J (2010). Hemodialysis-induced release of hemoglobin limits nitric oxide bioavailability and impairs vascular function. J Am Coll Cardiol.

[27] Nakano C, Morimoto S, Nakahigashi M, Kusabe M, Ueda H, Someya K, Ichihara A, Iwasaka T, Shiojima I (2015). The relationships between visit-to-visit blood pressure variability and renal and endothelial function in chronic kidney disease. Hypertension research : official journal of the Japanese Society of Hypertension.

[28] Khaira A, Mahajan S, Kumar A, Saraya A, Tiwari SC, Prakash S, Gupta A, Bhowmik D, Agarwal SK (2011). Endothelial function and oxidative stress in chronic kidney disease of varying severity and the effect of acute hemodialysis. Ren Fail.

[29] Chen J, Hamm LL, Mohler ER, Hudaihed A, Arora R, Chen CS, Liu Y, Browne G, Mills KT, Kleinpeter MA (2015). Interrelationship of multiple endothelial dysfunction biomarkers with chronic kidney disease. PLoS One.

[30] Hirata Y, Sugiyama S, Yamamoto E, Matsuzawa Y, Akiyama E, Kusaka H, Fujisue K, Kurokawa H, Matsubara J, Sugamura K (2014). Endothelial function and cardiovascular events in chronic kidney disease. Int J Cardiol.

[31] Tabata N, Hokimoto S, Akasaka T, Arima Y, Sakamoto K, Yamamoto E, Tsujita K, Izumiya Y, Yamamuro M, Kojima S (2016). Differential impact of peripheral endothelial dysfunction on subsequent cardiovascular events following percutaneous coronary intervention between chronic kidney disease (CKD) and non-CKD patients. Heart Vessel.

[32] Coffman JD (1994). Effects of endothelium-derived nitric oxide on skin and digital blood flow in humans. Am J Phys.

[33] Nohria A, Gerhard-Herman M, Creager MA, Hurley S, Mitra D, Ganz P (2006). Role of nitric oxide in the regulation of digital pulse volume amplitude in humans. J Appl Physiol.

[34] Joannides R, Haefeli WE, Linder L, Richard V, Bakkali EH, Thuillez C, Luscher TF (1995). Nitric oxide is responsible for flow-dependent dilatation of human peripheral conduit arteries in vivo. Circulation.

[35] Moerland M., Kales A. J., Schrier L., van Dongen M. G. J., Bradnock D., Burggraaf J. (2012). Evaluation of the EndoPAT as a Tool to Assess Endothelial Function. International Journal of Vascular Medicine.

[36] Hamburg NM, Keyes MJ, Larson MG, Vasan RS, Schnabel R, Pryde MM, Mitchell GF, Sheffy J, Vita JA, Benjamin EJ (2008). Cross-sectional relations of digital vascular function to cardiovascular risk factors in the Framingham heart study. Circulation.

[37] Brant LC, Hamburg NM, Barreto SM, Benjamin EJ, Ribeiro AL (2014). Relations of digital vascular function, cardiovascular risk factors, and arterial stiffness: the Brazilian longitudinal study of adult health (ELSA-Brasil) cohort study. J Am Heart Assoc.

[38] Akiyama E, Sugiyama S, Matsuzawa Y, Konishi M, Suzuki H, Nozaki T, Ohba K, Matsubara J, Maeda H, Horibata Y (2012). Incremental prognostic significance of peripheral endothelial dysfunction in patients with heart failure with normal left ventricular ejection fraction. J Am Coll Cardiol.

[39] Matsuzawa Y, Sugiyama S, Sumida H, Sugamura K, Nozaki T, Ohba K, Matsubara J, Kurokawa H, Fujisue K, Konishi M (2013). Peripheral endothelial function and cardiovascular events in high-risk patients. J Am Heart Assoc.

